# Anophthalmia and microphthalmia

**DOI:** 10.1186/1750-1172-2-47

**Published:** 2007-11-26

**Authors:** Amit S Verma, David R FitzPatrick

**Affiliations:** 1 MRC Human Genetics Unit, Edinburgh, UK

## Abstract

Anophthalmia and microphthalmia describe, respectively, the absence of an eye and the presence of a small eye within the orbit. The combined birth prevalence of these conditions is up to 30 per 100,000 population, with microphthalmia reported in up to 11% of blind children. High-resolution cranial imaging, post-mortem examination and genetic studies suggest that these conditions represent a phenotypic continuum. Both anophthalmia and microphthalmia may occur in isolation or as part of a syndrome, as in one-third of cases. Anophthalmia/microphthalmia have complex aetiology with chromosomal, monogenic and environmental causes identified. Chromosomal duplications, deletions and translocations are implicated. Of monogenic causes only *SOX2 *has been identified as a major causative gene. Other linked genes include *PAX6*, *OTX2*, *CHX10 *and *RAX*. *SOX2 *and *PAX6 *mutations may act through causing lens induction failure. *FOXE3 *mutations, associated with lens agenesis, have been observed in a few microphthalmic patients. *OTX2, CHX10 *and *RAX *have retinal expression and may result in anophthalmia/microphthalmia through failure of retinal differentiation. Environmental factors also play a contributory role. The strongest evidence appears to be with gestational-acquired infections, but may also include maternal vitamin A deficiency, exposure to X-rays, solvent misuse and thalidomide exposure. Diagnosis can be made pre- and post-natally using a combination of clinical features, imaging (ultrasonography and CT/MR scanning) and genetic analysis. Genetic counselling can be challenging due to the extensive range of genes responsible and wide variation in phenotypic expression. Appropriate counselling is indicated if the mode of inheritance can be identified. Differential diagnoses include cryptophthalmos, cyclopia and synophthalmia, and congenital cystic eye. Patients are often managed within multi-disciplinary teams consisting of ophthalmologists, paediatricians and/or clinical geneticists, especially for syndromic cases. Treatment is directed towards maximising existing vision and improving cosmesis through simultaneous stimulation of both soft tissue and bony orbital growth. Mild to moderate microphthalmia is managed conservatively with conformers. Severe microphthalmia and anophthalmia rely upon additional remodelling strategies of endo-orbital volume replacement (with implants, expanders and dermis-fat grafts) and soft tissue reconstruction. The potential for visual development in microphthalmic patients is dependent upon retinal development and other ocular characteristics.

## Disease names

Anophthalmia (OMIM 206900), Microphthalmia (OMIM 309700)

## Synonyms

Anophthalmos, microphthalmos, nanophthalmos, nanophthalmia

## Definition and diagnostic criteria

The mean maximum axial lengths in the neonatal and adult human eye are approximately 17 and 23.8 mm respectively. Most of the post-natal growth of the eye occurs within the first three years with posterior segment expansion accounting for over 90% of post-natal growth. The International Clearinghouse for Birth Defects Monitoring Systems defines anophthalmia and microphthalmia as "anophthalmos/microphthalmos: apparently absent or small eyes. Some normal adnexal elements and eyelids are usually present. In microphthalmia, the corneal diameter is less than 10 mm, and the antero-posterior diameter of the globe is less than 20 mm" [1].

## Epidemiology

The birth prevalence of anophthalmia and microphthalmia has been generally estimated to be 3 and 14 per 100,000 population respectively, although other evidence puts the combined birth prevalence of these malformations at up to 30 per 100,000 population [2,3]. Epidemiological data suggests risk factors for these conditions are maternal age over 40, multiple births [4,5], infants of low birth weight and low gestational age [6]. There is no predilection with regards to race or gender [4,5]. Both anophthalmia and microphthalmia are more commonly bilateral; the exception appears to be isolated microphthalmia, which is usually unilateral [5]. Microphthalmia is reported in 3.2 – 11.2% of blind children [7].

## Clinical description

**Figure 1:**
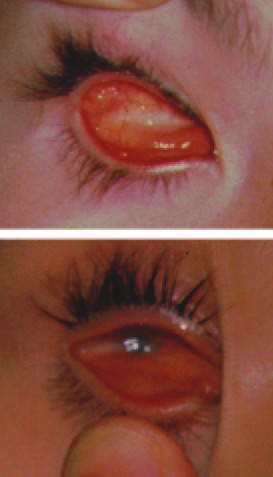
Clinical appearance of anophthalmia (upper picture) and microphthalmia (lower picture).

Anophthalmia refers to the absence of ocular tissue in the orbit. In the absence of clinically apparent ocular tissue, histological sectioning has shown residual neuroectoderm in some cases and hence terms such as 'true anophthalmia', 'clinical anophthalmia' and 'extreme microphthalmia' may in fact refer to what is in reality a phenotypic range between anophthalmia and microphthalmia (figure 1). Clinically it seems reasonable to use the term microphthalmia for an eye with axial length two standard deviations below that of the population age-adjusted mean; this typically correlates to an axial length below 21 mm in adult eyes. Simple microphthalmia refers to a structurally normal, small eye, and has been used interchangeably with 'nanophthalmia' (though the latter is particularly used when referring to a small eye with microcornea, axial length <18 mm, and = 8D hypermetropia). The increased thickness of the sclera in these eyes and the subsequent changes in blood flow are believed to be responsible for the increased incidence of uveal effusions and choroidal detachments seen. Microphthalmia may also be associated with other ocular disorders, in which case it is termed complex microphthalmia. These ocular disorders may affect the anterior segment (for example, sclerocornea and Peters anomaly) and/or the posterior segment (for example, persistent hyperplastic primary vitreous and retinal dysplasias). Both anophthalmia and microphthalmia can occur in isolation or be syndromic, as in about one-third of cases (see additional files 1 and 2 for a review of syndromes associated with anophthalmia and microphthalmia respectively). Learning disabilities are seen in approximately one-fifth of cases [2]. Complex microphthalmia, in particular, exhibits wide phenotypic variability.

## Aetiology

The precise pathogenesis of anophthalmia and microphthalmia remains unknown. Mann [8] suggested anophthalmia has its genesis early in gestation as a result of failure of development of the anterior neural tube (*secondary *anophthalmia) or optic pit(s) to enlarge and form optic vesicle(s) (*primary *anophthalmia). A third category, *consecutive *or *degenerative *anophthalmia was applied to cases where optic vesicles have degenerated and disappeared subsequent to formation. Observations of optic nerves, chiasm, and/or tracts with anophthalmia may indicate the regression of a partially developed eye rather than aplasia of the optic vesicle(s), a view supported by observations in an apparently anophthalmic orbit of extraocular muscle insertion into a fibrous mass, possibly representing an aborted eye [9]. Following observations that the posterior segment of microphthalmic eyes are more affected than the anterior, Weiss and colleagues [10,11] suggested that post-natal ocular growth is crucial and speculated that decreased size of the optic cup, altered proteoglycans in the vitreous, low intraocular pressure and abnormal growth factor production may all or in part have a bearing on the pathogenesis of simple microphthalmia; whilst inadequate production of secondary vitreous may result in complex microphthalmia. Some cases of microphthalmia may be associated with a cyst; these are believed to result from failure of the optic fissure to close [12].

Epidemiological studies have predicted both heritable and environmental factors in causing anophthalmia and microphthalmia. This review focuses on heritable causes as the evidence for environmental causes is both more circumstantial and accounts for a smaller proportion of cases. Chromosomal duplications, deletions and translocations have been implicated in both anophthalmia and microphthalmia, and are typically associated with characteristic syndromes (table 1). Of monogenic causes (table 2 shows selected genes with mutations linked to anophthalmia/microphthalmia), only *SOX2 *has to date been identified as a major causative gene for anophthalmia/microphthalmia. Cytogenetic studies placed the locus at 3q26.3, and *de novo *heterozygous loss-of-function point mutations have been shown to account for 10–20% of severe bilateral anophthalmia/microphthalmia [13], the most common phenotype being bilateral anophthalmia. The '*SOX2 *anophthalmia syndrome' encompasses sclerocornea, cataracts, persistent hyperplastic primary vitreous and optic disc dysplasia as well as non-ocular features like mental retardation, neurological abnormalities, facial dysmorphisms, post-natal growth failure, oesophageal pathology and anomalies of male genitalia [14,15].

**Table 1:** Chromosomal abnormalities associated with anophthalmia/microphthalmia [55,7].

**Chromosomal Abnormality**	**Other Features**
Duplication 3q syndrome (3q21-ter dup)	Learning difficulties, growth deficiency, hypertrichosis, craniosynostosis, cardiac defects, chest deformities, genital abnormalities, umbilical hernia
4p- (Wolf-Hirschhorn syndrome)	Growth deficiency, microcephaly, ocular hypertelorism, cranial asymmetry, learning difficulties, epilepsy, cleft lip/palate, anterior segment dysgenesis
Duplication 4p syndrome	Learning difficulties, epilepsy, growth deficiency, obesity, microcephaly, characteristic faces, genital abnormalities, kyphoscoliosis
Deletion 7p15.1-p21.1	Cryptophthalmos, cleft lip/palate, choanal atresia
Trisomy 9 mosaic syndrome	Joint contractures, congenital heart defects, prenatal growth deficiency, learning difficulties, micrognathia, kyphoscoliosis
Duplication 10q syndrome	Ptosis, short palpebral fissures, camptodactyly, learning difficulties, prenatal growth deficiency, microcephaly, heart and kidney malformations
13q-, 13 ring	Microcephaly, learning difficulties, bilateral retinoblastoma, cardiac defects, hypospadias, cryptorchidism
Trisomy 13 (Patau syndrome)	Holoprosencephaly, moderate microcephaly, coloboma, retinal dysplasia, cyclopia, cleft lip/palate, cardiac defects, genital abnormalities, 86% die within one year.
Deletion 14q22.1-q23.2	Pituitary hypoplasia.
18q-	Midface hypoplasia, small stature, learning difficulties, hypotonia, nystagmus, conductive deafness, microcephaly, midface hypoplasia, genital abnormalities
Trisomy 18 (Edwards syndrome)	Polyhydramnios, single umbilical artery, small placenta, low foetal activity, learning difficulties, hypertonicity, hypoplasia of skeletal muscle, subcutaneous, adipose tissue, prominent occiput, low-set malformed auricles, micrognathia, cardiac defects
Triploidy syndrome	Large placenta with hydatidiform changes, growth deficiency, syndactyly, congenital heart defects, brain anomalies/holoprosencephaly

**Table 2:** Ocular phenotypes associated with gene mutations linked to anophthalmia/microphthalmia.

**Gene**	**Locus (Inheritance)**	**Major (and selected less common) Human Ocular Phenotype(s)**	**OMIM [54]**
*SOX2*	3q26.3-q27 (AD)	Anophthalmia/microphthalmia	184429
*PAX6*	11p13 (AD)	Aniridia, (Peters anomaly, autosomal dominant keratopathy, foveal hypoplasia, optic nerve malformations, anophthalmia)	607108
*OTX2*	14q22 (AD)	Anophthalmia/microphthalmia, (retinal dysplasia, optic nerve malformations)	600037
*RAX*	18q21.3 (AR)	Anophthalmia/microphthalmia	601881
*CHX10*	14q24.3 (AR)	Microphthalmia	142993
*FOXE3*	1p32	Anterior segment dysgenesis, congenital primary aphakia	601094
*CRYBA4*	22q11.2-q13.1 (AD)	Autosomal dominant cataract, (microphthalmia)	123631

*PAX6*, on chromosome 11p13, has been studied more extensively than most other eye genes. In humans, heterozygous loss-of-function mutations typically produce aniridia (OMIM 106210), a congenital panocular malformation associated with severe visual impairment; however *PAX6 *was also the first gene implicated in human anophthalmia [16]. Although *PAX6 *mutations are an extremely rare cause of anophthalmia, there has recently been interest in a possible co-operative role between PAX6 and SOX2. Kondoh and colleagues [17] have shown that PAX6 and SOX2 co-bind to a regulatory element driving lens induction in the chick, which suggests that lens induction failure could be responsible for microphthalmia in patients with mutations in these genes [9]. PAX6 and SOX2 interactions have since been shown to also drive lens induction in mammals through their action on the γ-crystallin gene (V van Heyningen, personal communication). Ultrasound bimicroscopy studies are required to determine if aphakia is commonly associated in microphthalmic *SOX2 *cases. As expected with genes expressed in the developing brain, patients with inherited *PAX6 *and *SOX2 *mutations exhibit CNS malformations in addition to dominantly inherited anophthalmia/microphthalmia [18,9]. Interestingly mutations within the *FOXE3 *gene (on chromosome 1p32), associated with congenital primary aphakia (OMIM 610256), were observed in three siblings with microphthalmia; in all three cases the phenotype was believed to be secondary to lens agenesis [19].

Mutations in three genes with retinal expression are associated with anophthalmia/microphthalmia, possibly through failure of retinal differentiation. Heterozygous loss-of-function mutations of *OTX2 *(on chromosome 14q22, autosomal dominant inheritance) have been shown to be associated with a wide range of ocular disorders from anophthalmia and microphthalmia to retinal defects. CNS malformations and mental retardation are common in patients with *OTX2 *mutations [20,9]. *RAX*, located on chromosome 18q21.32, is linked to about 2% of inherited anophthalmia/microphthalmia [21]. Similarly, *CHX10 *mutations (chromosome 14q24.3) account for about 2% of isolated microphthalmia [22]; mutations in both genes characteristically presenting with recessively inherited phenotypes.

Two syndromes with broad phenotypes have been described recently in association with anophthalmia. *GLI2 *mutations had originally been described in the context of holoprosencephaly and polydactyly, however there has been a case reporting a missense mutation in a patient with asymmetrical genu and callosal agenesis co-existing with anophthalmia, thereby extending the phenotype [23]. Anophthalmia with congenital heart defects, pulmonary abnormalities, diaphragmatic hernia and learning difficulties have been described in patients with mutations of the *STRA6 *gene [24]. Our knowledge of genes associated with microphthalmia has also increased; complex microphthalmia in association with genetic cataracts has been attributed to mutations in the *CRYBA4 *gene [25]. In addition to these putative genes, several loci have been identified with autosomal dominant microphthalmia mapping to 15q12-15 [26], autosomal recessive microphthalmia mapping to 14q32 [27,28] and X-linked anophthalmia mapping to Xq27-28 [29].

Over the past several years, there has been an increased awareness of environmental factors associated with anophthalmia/microphthalmia. In 1993, the UK media reported clusters of anophthalmia and microphthalmia patients, speculating that these conditions may be connected to the pesticide Benomyl. Studies specifically designed to look at this issue found no definitive causal link [30-33]. The strongest evidence for environmental causes is for gestational-acquired infections, with rubella, toxoplasmosis, varicella and cytomegalovirus implicated [30,34]. Other viruses in the herpes-zoster family have also been linked, as have parvovirus B19, influenza virus, and coxsackie A9 [35,36]. Non-infectious causes have been postulated and include maternal vitamin A deficiency [37], fever, hyperthermia, exposure to X-rays, solvent misuse and exposure to drugs like thalidomide, warfarin and alcohol [30].

## Diagnostic methods

The diagnosis is usually based upon clinical and imaging criteria, and may be confirmed on histology if post-mortem is performed. Establishing a specific cause involves undertaking a comprehensive medical history, physical examination, family history, karyotyping and molecular genetic testing, imaging, renal ultrasonography, and audiology.

### Ophthalmological assessment

Anophthalmia can potentially be a difficult clinical diagnosis to make. Microphthalmia is usually diagnosed by inspection and palpation of the eye through the lids. The diagnosis is aided by measurements of corneal diameter, which ranges from 9–10.5 mm in neonates and 10.5–12 mm in adults. Microphthalmia with cyst usually presents with lower lid bulging. Electrodiagnostic tests may be valuable, particularly in cases of microphthalmia where retinal development has been unaffected. Eye examination of both parents should be undertaken and a careful family history of eye anomalies sought.

### Paediatric and clinical genetics assessment

Because of the wide phenotypic spectrum associated with anophthalmia/microphthalmia, it is vital to assess these patients within multi-disciplinary teams that include paediatricians and clinical geneticists. Further investigations are dependent upon the clinical picture. If no syndrome is identified in infancy, further examination after another three or four years is desirable as many syndromes become more apparent by this age.

### Imaging

Ultrasound is most commonly used to determine the length of the globe in microphthalmic eyes.

**Figure 2:**
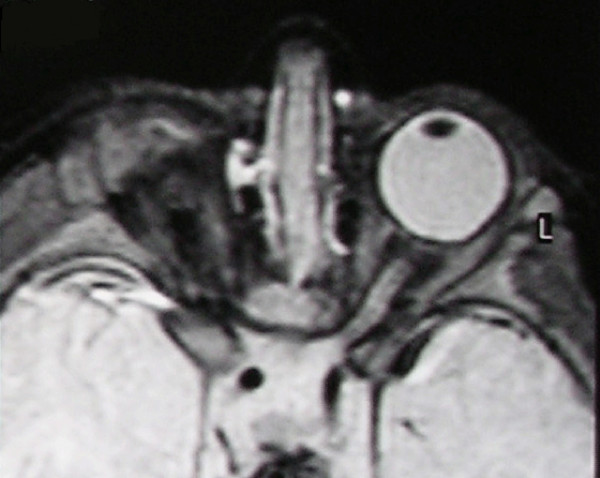
**T2-weighted MR scan of a patient with unilateral anophthalmia**. Note the presence of amorphous tissue and structures resembling extraocular muscles within the anophthalmic right orbit. The right optic nerve/chiasm junction appears attenuated rather than absent suggesting possible residual optic nerve neural tissue.

CT and MR scans facilitate the diagnosis of anophthalmia. Both scans show the absence of a globe within the orbit although soft amorphous tissue may be discerned (intermediate T1 signal intensity and low T2 signal intensity on MR scan, intermediate density on CT scan). Neural tissue forming the visual pathway and extraocular muscles are variably present (figure 2) [38-40]. Orbital dimensions and volume are both reduced [38]. Simple microphthalmia shows as a normal albeit small globe, with normal signal/density characteristics of lens and vitreous, in a smaller orbit than normal.

## Differential diagnosis

*Cryptophthalmos *refers to completely fused eyelid margins, without lashes. These cases can be associated with both microphthalmia and microcornea. It is often bilateral and may be syndromic.

*Cyclopia *(total) and *synophthalmia *(partial) represent degrees of fusion of the optic vesicles thereby preventing the development of separate eyes. They correspond to neural maldevelopments incompatible with life.

In contrast to microphthalmia with cyst, which results from failure of the optic fissure to close, a *congenital cystic eye *may develop from failure of the optic vesicle to invaginate [12]. At birth, the cystic eye may resemble anophthalmia, however with post-natal expansion, a bulge may appear behind the eyelids.

## Genetic counselling

Genetic counselling is challenging both from the perspective of the extensive range of genes responsible for anophthalmia/microphthalmia and the wide variation in phenotypic expression. Only *SOX2 *has thus far been identified as a major anophthalmia/microphthalmia gene, with mutations primarily arising *de novo*. The picture is further complicated by observations of phenotypically normal parents carrying loss of function *SOX2 *or *OTX2 *mutations [41,20]. Mosaicism and/or variable penetrance render prediction of recurrence risk difficult in these monogenic anophthalmia/microphthalmia cases. In general, if the mode of inheritance can be identified, then appropriate counselling is indicated. The empiric risk to siblings without a clear aetiology or family history is 10–15%, assuming inheritance accounts for half of cases with the other half occurring sporadically [7]. Chromosomal abnormalities associated with anophthalmia/microphthalmia tend to be associated with distinct co-morbidities and give rise to specific syndromes. If a patient has a numerical chromosomal abnormality, the parents can be expected to be entirely normal whilst siblings are at a slightly increased risk of having a similar chromosomal abnormality, with similar or dissimilar phenotype [7]. If a patient has a structurally unbalanced chromosomal constitution, the parents may have balanced chromosomal rearrangements and other siblings will be at a higher risk, though this will depend upon the specific rearrangement. If neither parent has any rearrangement, the risk to siblings is virtually negligible [7].

## Antenatal diagnosis

### Chromosome analysis

Cytogenetic studies are possible upon amniotic fluid foetal cells (usually withdrawn after 14 weeks of gestation) or on chorionic villus sampling specimens (at about 10 to 12 weeks). The power of these techniques in facilitating the pre-natal diagnosis of anophthalmia/microphthalmia was elegantly demonstrated by Guichet and colleagues (2004) [42]. In a foetus with severe intrauterine growth retardation and bilateral anophthalmia on a 24-week ultrasound scan, they demonstrated a 46, XX, del(3)(q26.3q28) interstitial deletion of the long arm of chromosome 3 on 650 band karyotype. FISH analysis confirmed the interstitial deletion of 3q27 encompassing the *SOX2 *locus.

### Ultrasonography

It is possible to detect anophthalmia/microphthalmia by early second trimester [43], though more recent reports place the limit at about 12 weeks with trans-vaginal ultrasound [44,45]. Foetal eyes are best scanned in the coronal, axial and corono-axial planes and appear as symmetrical structures on either side of the nose. Lenses appear as smooth circular lines with hypoechogenic content on axial and coronal views. Eye size can be measured upon visualising the maximum coronal or axial planes of the orbit, and compared against established eye growth charts [46,47].

### MRI

where available can be used to supplement ultrasonography.

## Management

### Conservative

Detectable retinal function may be present in microphthalmia cases, particularly those associated with *SOX2 *mutations. It is important to refract these eyes and treat any underlying amblyopia. In unilateral cases, the 'good' eye must be protected and any visual deficit managed appropriately.

### Surgical

Surgical management can form the mainstay of anophthalmia/microphthalmia treatment. The globe triples in volume between birth and adolescence. The growth of the bony orbit reflects growth of the globe [48]. Both congenital anophthalmia and microphthalmia result in a small volume orbit compared to age-matched controls [49], potentially leading to the appearance of hemifacial asymmetry. There is also evidence that enucleation (removal of the globe) produces a reduction in orbital volume in both children and adults [50,51]. Reconstructive strategies rely upon the simultaneous management of both soft tissue hypoplasia and asymmetric bone growth [52].

Treatment is usually started early to maximise the overall development of these children. Mild/moderate microphthalmia is generally managed conservatively with insertion of a conformer (like a prosthetic eye but not painted), periodically increasing in size to allow for growth of the orbit. Treatment for severe microphthalmia and anophthalmia are usually started within weeks of life using conformers to enlarge the palpebral fissure, conjunctival cul-de-sac and orbit [48]. Endo-orbital volume replacement using implants of progressively increasing size can be used to stimulate expansion of the developing bony orbit, usually after six months of age. Volume replacement using implants and expanders can also be supplemented by the use of dermis-fat grafts. Static orbital implants may need to be changed between three and five times before puberty and are associated with problems of wound dehiscence, extrusion or inadequate stimulation of bony growth. Expandable orbital implants were introduced as an efficacious means of stimulating bony growth and socket enlargement. Inflatable expanders are limited by difficulty maintaining orbital fixation for sustained expansion and controlling the direction of expansion, whilst self-expanding hydrogel spheres lose expansion force once fully hydrated. Orbital osteotomies are indicated in more severe cases [48,52]. Ocular prostheses are used when the orbit has developed adequately, and are changed regularly with further orbital expansion. Conjunctival sac and lid reconstruction may be beneficial to the overall cosmetic effect. Microphthalmia with cyst is often treated around the age of five permitting the ophthalmic surgeon to take advantage of the orbital expansion properties of the cyst until the orbit is about 90% of the adult volume, whilst allowing removal for cosmetic reasons at about the time the child starts school. Surgical excision with preceding decompression is commonly performed, the cyst may also be aspirated but the recurrence rate is higher [53].

## Prognosis

The potential for visual development depends upon the degree of retinal development and other ocular characteristics in microphthalmic patients. Therapy aims to maximise existing vision and enhance cosmetic appearances rather than improve sight.

## Unresolved issues

The aetiology of anophthalmia/microphthalmia underlies the entire developmental biology of ocular formation and remains a field where our knowledge is increasing exponentially. Despite the progresses made, much work is still needed to understand the processes underlying these complex diseases, which are a significant cause of childhood blindness. Even if these processes are elucidated in the future, novel therapeutic approaches to prevent these conditions from occurring could still be precluded by very early ocular development in the foetus.

## Abbreviations used

AD (autosomal dominant);

AR (autosomal recessive);

CNS (central nervous system);

CT (computerised tomography);

MR (magnetic resonance); 

MRI (magnetic resonance imaging);    

OMIM (Online Mendelian Inheritance in Man [54]).

## Competing interests

The author(s) declare that they have no competing interests.

## Authors' contributions

ASV drafted the manuscript and both authors subsequently revised the manuscript for intellectual content.

## Supplementary Material

Additional file 1 Syndromes associated with anophthalmia. A description of the clinical syndromes known to be associated with anophthalmia. This table also includes known (or postulated) genetic associations. Click here for file

Additional file 2 Syndromes associated with microphthalmia. A description of the clinical syndromes known to be associated with microphthalmia. This table also includes known (or postulated) genetic associations. Click here for file
