# Supplementary material for: Anophthalmia and microphthalmia
Source: Orphanet J Rare Dis. 2007 Nov 26;2:47. doi: 10.1186/1750-1172-2-47 (PMC2246098; doi:10.1186/1750-1172-2-47)
Supplement: Additional file 1 — Syndromes associated with anophthalmia. A description of the clinical syndromes known to be associated with anophthalmia. This table also includes known (or postulated) genetic associations. [file 1750-1172-2-47-S1.doc]

**Additional file 1.** Syndromes associated with anophthalmia.

| **Syndrome** | **Inheritance** | **Locus (Gene)** | **Characteristics in Addition to Anophthalmia** | **OMIM [54]** |
| --- | --- | --- | --- | --- |
| Fryns | AR | Unknown | Orofacial clefting, uterine abnormalities, ear abnormalities, neural tube defects, microphthalmia | 229850 |
| Lenz microphthalmia | X-linked | Xq27-q28 (*ANOP1*),  Xp11.4-p21.2 (*BCOR*) | Microphthalmia, learning difficulties, limb abnormalities, microcephaly, orofacial clefting, tooth and skeletal anomalies, deafness, microgenitalia | 309800 |
| Matthew-Wood | ? AR | 15q23-q25.1 (*STRA6)* | Pulmonary hypoplasia | 601186 |
| Oculocerebrocutaneous (Delleman) syndrome | AR | Unknown | Orbital cysts, focal dermal hypoplasia, cerebral malformations, cleft lip/palate in 15% | 164180 |
| Waardenburg-Anophthalmia | AR | Unknown | Syndactyly / oligodactyly, learning difficulties, skeletal anomalies | 206920 |
