# Supplementary material for: Anophthalmia and microphthalmia
Source: Orphanet J Rare Dis. 2007 Nov 26;2:47. doi: 10.1186/1750-1172-2-47 (PMC2246098; doi:10.1186/1750-1172-2-47)
Supplement: Additional file 2 — Syndromes associated with microphthalmia. A description of the clinical syndromes known to be associated with microphthalmia. This table also includes known (or postulated) genetic associations. [file 1750-1172-2-47-S2.doc]

**Additional file 2. Syndromes associated with microphthalmia.**

| **Syndrome** | **Inheritance** | **Locus (Gene)** | **Characteristics in Addition to Microphthalmia** | **OMIM [54]** |
| --- | --- | --- | --- | --- |
| Aicardi syndrome | X-linked dominant | Xp22 | Agenesis of corpus callosum, anophthalmia, chorioretinal abnormality, infantile spasms, microcephaly, cleft lip / palate, rib/vertebrae abnormalities, brain abnormalities, neoplasia, precocious puberty, learning difficulties, lethal in males | 304050 |
| Arhinia, choanal atresia, and microphthalmia | AD | Unknown | Complete absence of nose, choanal atresia, cleft palate | 603457 |
| Branchio-oculo-facial syndrome (BOFS) | AD | Unknown | Developmental delay, microcephaly, anophthalmia, coloboma, renal malformations, cataract, cleft lip / palate, dental anomalies, branchial arch anomalies, infra-auricular or cervical skin defects | 113620 |
| CHARGE syndrome | AD | 7q21.11 (semaphorin-3E), 8q12.1 (*CHD7*) | Coloboma, heart defect, choanal atresia, growth and developmental retardation, genital and ear abnormalities | 214800 |
| Cerebro-oculo-facio-skeletal syndrome (COFS) | AR | 10q11 (*ERCC6*) | Hypotonia, cataract, microcephaly, brain malformations, blepharophimosis, kyphoscoliosis, osteoporosis, kidney defects, failure to thrive, flexion contractures | 214150 |
| Coloboma-obesity-hypogenitalism-mental retardation syndrome | AD | Unknown | Cataract, coloboma, obesity, hypogenitalism, learning difficulties | 601794 |
| Dextrocardia with unusual facies | AR | Unknown | Dextrocardia, dysmorphic facies, normal growth, anophthalmia | 221950 |
| Facio-thoraco-genital syndrome | AR | Unknown | Characteristic facies, pectus excavatum, micrognathia, genital abnormalities | 227320 |
| Focal Dermal Hypoplasia (Goltz-Gorlin syndrome) | X-linked Dominant | Xp22 | Atrophy and linear pigmentation of skin, multiple mucous membrane papillomas, colobomas, digital anomalies, hypoplastic teeth, learning difficulties, lethal in males | 305600 |
| Frontonasal dysplasia syndrome | Possibly sporadic |  | Hypertelorism, lateral displacement of inner canthi, deficit in midline frontal bone, absence of corpus callosum | 136760 |
| GOMBO syndrome | AR | 3p or 22q | Microcephaly, digital abnormalities, delayed growth at puberty, learning difficulties | 233270 |
| Hallermann-Streiff syndrome (Francois dyscephalic syndrome) | Possibly sporadic |  | Cataracts, coloboma, hypotrichosis, postnatal growth retardation, brachycephaly, malar hypoplasia, dental abnormalities, atrophy of skin, bony abnormalities | 234100 |
| Incontinentia Pigmenti | X-linked Dominant | Xq28 (*IKBKG*) | Skin changes, microcephaly, learning difficulties, anophthalmia, retinal dysplasia and vascularisation, male lethal | 308300 |
| Kapur-Toriello syndrome | AR | Unknown | Coloboma, learning difficulties, congenital heart defects, cleft lip / palate, intestinal malrotation, renal displacement | 244300 |
| Kenney-Caffey | ? AD  ? X-linked | Unknown | Short stature, bone abnormalities, hypocalcaemia, hypophosphataemia | 127000 |
| Lenz microphthalmia syndrome | X-linked | Xq27-q28 (*ANOP1*),  Xp11.4-p21.2 (*BCOR*) | Learning difficulties, distal limb abnormalities, microcephaly, orofacial clefting, tooth and skeletal anomalies, hearing loss, genitourinary malformations, anophthalmia, colobomas | 309800 |
| Macrosomia with microphthalmia, lethal | AR | Unknown | Macrosomia, early infant death from overwhelming infection | 248110 |
| Meckel syndrome, type 1–3 | AR | 17q23 (*MKS1*), 8q24 (*MKS3*), 11q13 (*MKS2*) | Renal cysts, developmental anomalies of the CNS, hepatic duct dysplasia and cysts, polydactyly | 249000 |
| Microphthalmia with cataract | AD | 16p13.3 | Congenital cataract | 156850 |
| Microphthalmia with cyst, limb anomalies, bilateral facial clefts | Unknown | Unknown | Features similar to incomplete Waardenburg ophthalmo-acromelic syndrome / cerebro-oculonasal syndrome / craniotelencephalic dysplasia | 607597 |
| Microphthalmia, dermal aplasia, sclerocornea (MIDAS) syndrome | X-linked  Dominant | Xp22.31 | Cardiac abnormalities, skin atrophy, linear skin defects, sclerocornea, orbital cysts, Peter anomaly, anophthalmia, lethal in utero in males | 309801 |
| Microcephaly, microphthalmia, ectrodactyly of lower limbs, prognathism (MMEP) | ? AD  ? Sporadic | 6q21 (*SNX3*) | Microcephaly, ectrodactyly of the lower limbs, prognathism | 601349 |
| Microphthalmia with coloboma 2 | AD | 15q12-q15 | Coloboma, microcornea. Variable expressivity with some showing only Peters anomaly, optic nerve agenesis | 605738 |
| Mucolipidosis III | AR | 12q23.3 (*GNPTAB*) | Short stature, corneal clouding, retinopathy, bone abnormalities, aortic insufficiency, learning difficulties, skin thickening | 252600 |
| Nance-Horan (cataract dental) syndrome | X-linked | Xp22.13  (*NHS)* | Cataract, microcornea, learning difficulties, long narrow face, prominent nose, short fourth metacarpals | 302350 |
| Norrie Disease | X linked | Xp11.4 (*NDP*) | Retinal dysplasia and abnormalities | 310600 |
| Oculo-auriculo-vertebral dysplasia (Goldenhar syndrome) | Most are probably sporadic, few AD | 14q32 | Unilateral deformity of external ear, vertebral anomalies, cleft lip / palate, hearing loss, facial nerve palsy, epibulbar dermoids, lid colobomas, Duane syndrome | 164210 |
| Oculodentodigital Syndrome | AD | 6q21–23.2 (*GJA1*) | Characteristic facies, microcephaly, syndactyly, hypo- and aplasia middle phalanges, dental abnormalities, glaucoma, cataract, optic atrophy | 164200 |
| Oculo-dento-osseous dysplasia, recessive | AR | Unknown | Microcornea, iris dysplasia, persistent hyperplastic primary vitreous, malformed teeth, syndactyly, widened long bones | 257850 |
| Oculo-facio-cardio-dental (OFCD) syndrome | X-linked dominant | Xp11.4 (*BCOR*) | Congenital cataract, long narrow face, cleft lip / palate, heart defects, dental anomalies, lethal in males | 300166 |
| Osteoporosis-pseudoglioma Syndrome | AR | 11q13.4 (*LRP5*) | Osteogenesis imperfecta, retinal dysplasia, persistent hyperplastic primary vitreous, corneal opacity, secondary glaucoma, pseudoglioma, hypotonia, ligamentous laxity | 259770 |
| Papillorenal (renal coloboma) syndrome | AD | 10q24.3-q25.1 *(PAX2)* | Coloboma, hypoplastic kidney | 120330 |
| Steinfeld Syndrome | AD | Unknown | Holoprosencephaly, hypoplasia of the forearm bones, renal dysplasia, cleft lip / palate, congenital heart disease, vertebral / rib anomalies | 184705 |
| Solitary median maxillary central incisor (SMM1) | AD | 7q36 (*SHH*) | Single central incisor, holoprosencephaly, short stature with growth hormone deficiency, coloboma, ectodermal dysplasia | 147250 |
| Walker-Warburg syndrome | AR | 9q34.1 (*POMT1*),  14q24.3  (*POMT2*),  19q13.3  (*FKRP*),  9q31  (*FCMD*) | Hydrocephalus, congenital muscular dystrophy, retinal dysplasia/congenital retinal detachment, anterior chamber anomalies, cataract, glaucoma, coloboma, Peters anomaly, persistent hyperplastic primary vitreous, cleft lip / palate, genitourinary anomalies, lissencephaly, lethal by one year of age | 236670 |
| Warburg Micro syndrome (WARBM) | AR | 2q21 (RAB3GAP) | Microcornea, cataract, abnormal ERG, optic atrophy, microcephaly, learning difficulties, truncal hypotonia, microgenitalia | 600118 |
